# Generalization of the Landauer Principle for Computing Devices Based on Many-Valued Logic

**DOI:** 10.3390/e21121150

**Published:** 2019-11-25

**Authors:** Edward Bormashenko

**Affiliations:** Department of Chemical Engineering, Engineering Sciences Faculty, Ariel University, Ariel 407000, Israel; edward@ariel.ac.il; Tel.: +972-074-729-68-63

**Keywords:** Landauer principle, binary logic, ternary logic, Landauer bound, trit

## Abstract

The Landauer principle asserts that “the information is physical”. In its strict meaning, Landauer’s principle states that there is a minimum possible amount of energy required to erase one bit of information, known as the Landauer bound $W=k_{B}Tln2$, where *T* is the temperature of a thermal reservoir used in the process and $k_{B}$ is Boltzmann’s constant. Modern computers use the binary system in which a number is expressed in the base-2 numeral system. We demonstrate that the Landauer principle remains valid for the physical computing device based on the ternary, and more generally, *N*-based logic. The energy necessary for erasure of one bit of information (the Landauer bound) $W=k_{B}Tln2$ remains untouched for the computing devices exploiting a many-valued logic.

## 1. Introduction

Modern computers use the binary system, whereby a number is expressed in the base-2 numeral system. The base-2 numeral system is a positional notation with a radix of 2. Each digit is referred to as a bit. The base-2 is ubiquitous in computing devices because of its straightforward implementation in digital electronic circuitry using binary logic gates. However, one of the first computing machines was based on the ternary logic. In 1840, Thomas Fowler, a self-taught English mathematician and inventor, created a unique ternary mechanical calculating machine, completely manufactured of wood [1]. Ternary logic based computers based in the “trit” unit of information were successfully developed in Soviet Union by Nicolay Brousentsov [2]. The Setun computer, based on the ideas of ternary logic, ternary symmetrical number system and ternary memory element (“flip-flap-flop”) was designed in 1958 in Moscow University [2,3]. In principle, computer may be based on a many-valued logics, exposed in recent years to a growing interest due to the fundamental aspects and numerous applications [4,5]. Ternary computer TERNAC was reported in 1973 by Frieder et al. in Reference [6]. 

The present paper does not come into the mathematical details of the ternary (or another) many-valued logics, but extends the Landauer principle to the erasing of the information by the computing machine, based on the many-valued logics. Informational theory is usually supplied in a form that is independent of any physical realization. In contrast, Rolf Landauer, in his papers, argued that “information is physical” and has an energy equivalent [7,8,9]. It may be stored in physical systems, such as books and memory chips, and it is transmitted by physical devices exploiting electrical or optical signals [6,7,8]. Therefore, he concluded, it must obey the laws of physics, and first and foremost, the laws of thermodynamics. The Landauer principle [7,8,9] established the energy equivalent of information and remains a focus of investigations in the last decade [10,11,12,13,14,15,16,17,18]. In its strictest, tightest, and simplest meaning, the Landauer principle states that the erasure of one bit of information requires a minimum energy cost equal to $k_{B}Tln2$, where *T* is the temperature of a thermal reservoir used in the process and $k_{B}$ is Boltzmann’s constant [7,8,9,10,11,12,13,14]. The Landauer principle is usually demonstrated with the computers, based on the binary logic. We demonstrate how it may be extended to devices that exploit a many-valued logics. 

## 2. Discussion

**Figure 1:**
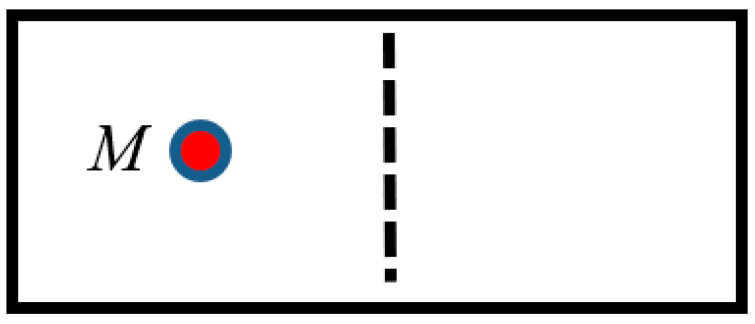
Finding of the particle *M* in the certain (left or right) half of the chamber corresponds to the recording of 1 bit of information.

**Figure 2:**
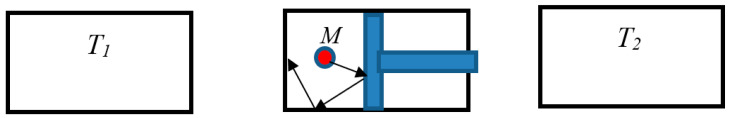
Sketch of the minimal single-particle thermal machine is depicted. Particle *M* moves the piston. The machine works between the hot (*T*_1_) and cold (*T*_2_) thermal reservoirs which may be finite. The conditions of “thermalization” (randomization) of the particle motion are discussed.

Consider the computing device exploiting a particle enclosed within a chamber (cylinder) divided by half by a partition, as shown in Figure 1. Finding the particle *M* in the certain (left or right) half of the chamber corresponds to the recording of 1 bit of information. When the partition is removed, the location of the particle is uncertain, and this corresponds to the erasure of 1 bit of information. The location of a particle on a certain half of the chamber corresponds to “1”, and the uncertain location of the particle corresponds to “0”, thus our particle-based computer works on the binary logical system. The work of this computer may be exemplified by the single-particle thermal engine, suggested by Leo Szilard in 1929 [19] and depicted in Figure 2. The smallest possible thermodynamic machine consists of a single particle of mass *m* in a closed cylinder, which has contact with a thermal reservoirs. Consider the “evergreen” Carnot cycle, performed by a minimal engine, is depicted in Figure 1 from an informational point of view [9,20,21]. At the first stage, the particle contacts the thermal reservoir (bath) *T*_1_ and undergoes a reversible isothermal expansion, which doubles its available volume [21]. Note, that the particle initially occupies the left side of the cylinder. Heat *k_B_T*_1_ *ln2* is drawn from the bath and work $k_{B}T_{1}ln2$ is extracted. This process is equivalent to the removal of the partition at the midpoint of the cylinder, thus, one bit of information is *erased*, if one bit finds particle *m* at a certain side (left in our case) of the cylinder, as shown in Figure 2 [10]. Thus, heat *k_B_T*_1_ *ln*2 spent by the thermal bath was exploited for erasure of 1 bit of information.

At the second stage our engine is exposed to the adiabatic expansion and the additional mechanical work is made. At this stage, the entropy of the working body and the thermal reservoir remain unchanged, and there is no informational change in both of them (thermal reservoir *T*_1_ is disconnected from the engine at this stage). At the next stage, the engine is connected to the thermal bath *T*_2_ and exerted to the reversible isothermal compression. A piston reversibly and isothermally compresses the space occupied by the particle *m* from full to half volume. One bit of information is recorded by the engine. Heat $Q_{2}=$ *k_B_T*_2_ *ln*2, is delivered to the heat bath, and work *kT*_2_ *ln*2 is consumed. At the last stage of the cycle the engine is disconnected from the reservoir *T*_2_ and the system is adiabatically heated to the temperature *T*_1_. No entropy and informational changes take place at this stage. The work of the minimal Carnot engine illustrates the Landauer principle: Recording/erasing one bit of information demands $k_{B}Tln2$ units of energy. 

The non-trivial problems of “thermalization” of the motion of the particle in the minimal Carnot engine are out of the scope of our paper [10,20,21]. The Carnot engine is fully reversible; actually, the erasure/recording of information is asymmetric and there may be no entropy cost to the acquisition of information, but the destruction of information does involve an irreducible entropy cost [21]. This erasure/recording asymmetry is essential [10,22]. However, it is not in the focus of the present paper. Note, that the efficiency of the engine equals $\eta =1-\frac{T_{2}}{T_{1}}$, as demonstrated in Reference [21]. This result is quite expected, due to the fact that the efficiency of the Carnot machine is insensitive to working substances in the engine and depends only on the temperatures of the thermal reservoirs [21].

**Figure 3:**
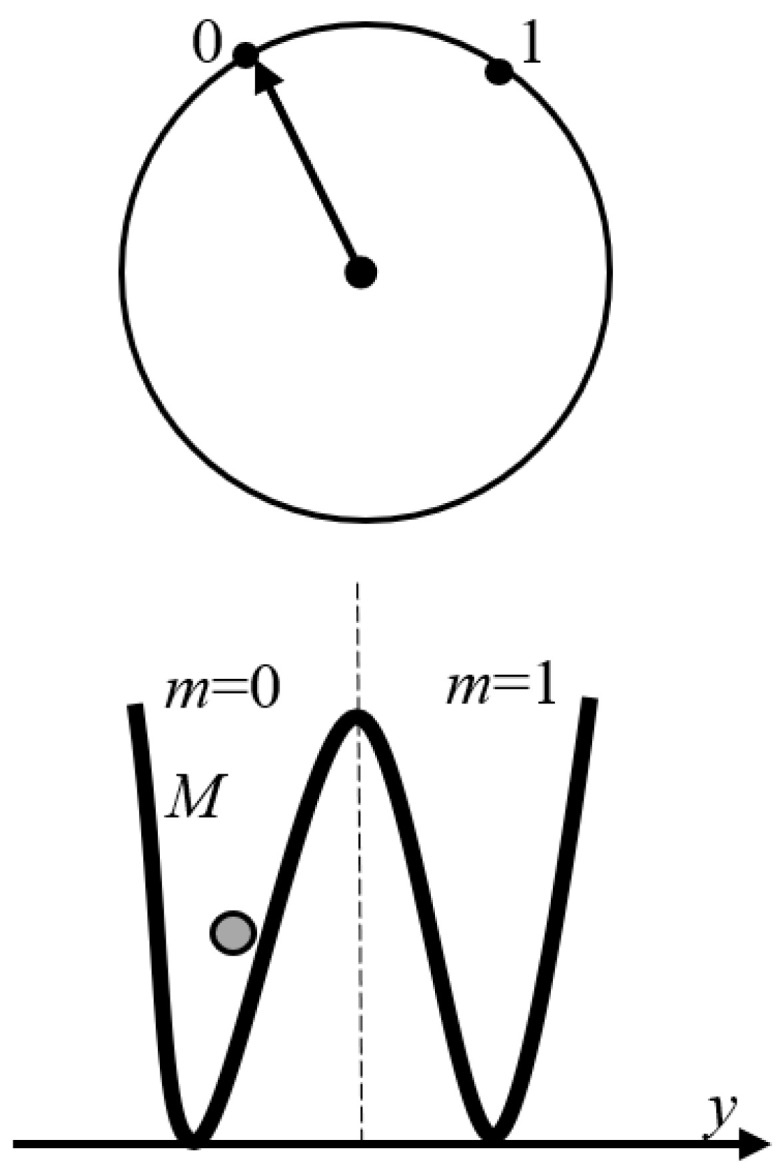
The qubit model of a memory exploiting a Brownian particle *M* in a symmetrical double-well potential with position *y* which can be stably trapped in either left or right well, corresponding to informational states m=0; m=1 (see Reference [9]).

The additional exemplification of the Landauer principle is supplied by the Brownian particle in a double-well potential, as shown in Figure 3 and discussed in detail in References [8,10]. When the barrier is much higher than the thermal energy, the particle will remain in either well for a long time [8,10,20]. Thus, the particle being in the left or right well can serve as the stable informational states, “0” and “1” of a bit. A Brownian particle trapped in either left or right well represents the informational states m=0 and m=1, as shown in Figure 3, where *m* is the parameter, characterizing the statistical state of the system. The average work *W* to change the statistical state of a memory from the state Ψ with the distribution $p_{m}$ to $\Psi^{\prime }$ with distribution $p_{m}^{\prime }$ is given by Equation (1a,b):

(1a) $$W\geq F\left(\Psi^{\prime }\right)-F\left(\Psi \right)$$

(1b) $$F\left(\Psi \right)=\underset{m}{\sum }p_{m}F_{m}+k_{B}T\underset{m}{\sum }p_{m}lnp_{m}$$

where $F_{m}=E_{m}-TS_{m}$ is the free energy of the conditional state [10]. For a symmetrical well and a random bit $p_{0}=p_{1}=\frac{1}{2}$, we immediately recover the Landauer bound $W=k_{B}Tln2$, as shown in Reference [10] and checked experimentally in References [23,24,25]. The colloidal particle in a double-well potential was used as a generic model of a one-bit memory in Reference [23]. The experimental verification of the Landauer principle was carried out with an optical tweezer, which trapped a silica bead (2 μm in diameter) at the focus of a laser beam [23]. It was demonstrated that that the mean dissipated heat saturated at the Landauer bound in the limit of long erasure cycles [23]. Reference [24] reports testing of the Landauer principle with a colloidal particle confined in a time-dependent, virtual potential which is created by a feedback trap. The extension of the Landauer principle to the quantum realm is carried out by using a crystal of molecular nanomagnets as a quantum spin memory, as demonstrated in Reference [25]. Employing a trapped ultracold ions enabled experimental validation of a quantum version of the Landauer principle [26]. In contrast, the experiment, which combinational logic realized with a micro-electromechanical cantilever, is reported in Reference [27]. The authors stated that the logical device can be operated with energy well below $k_{B}T$, at room temperature, if the operation is performed slowly enough and friction losses are minimized [27]. Thus, it was suggested that no fundamental energy limit need be associated with irreversible logic computation in general [27]. These results were criticized in Reference [28], in which it was shown that the approach, reported in Reference [27], neglects the dominant source of energy dissipation, namely, the charging energy of the capacitance of the input electrode, which totally dissipates during the full cycle of logic values [28]. The analysis of the asymmetrical potential well, performed in Reference [10], is out of the scope of our paper.

**Figure 4:**
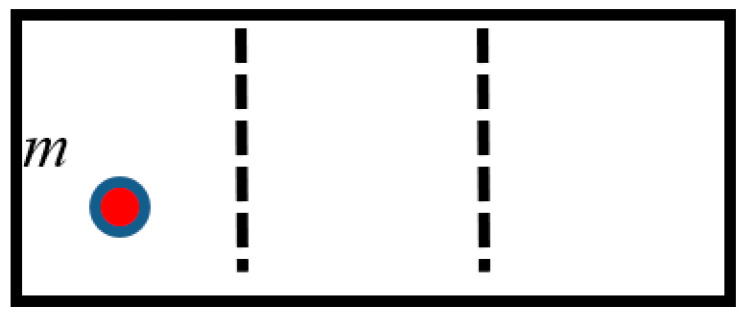
Finding of the particle *m* in the certain one-third part of the chamber corresponds to the recording of 1 bit of information. Thus, the “trit”-based computation becomes possible.

**Figure 5:**
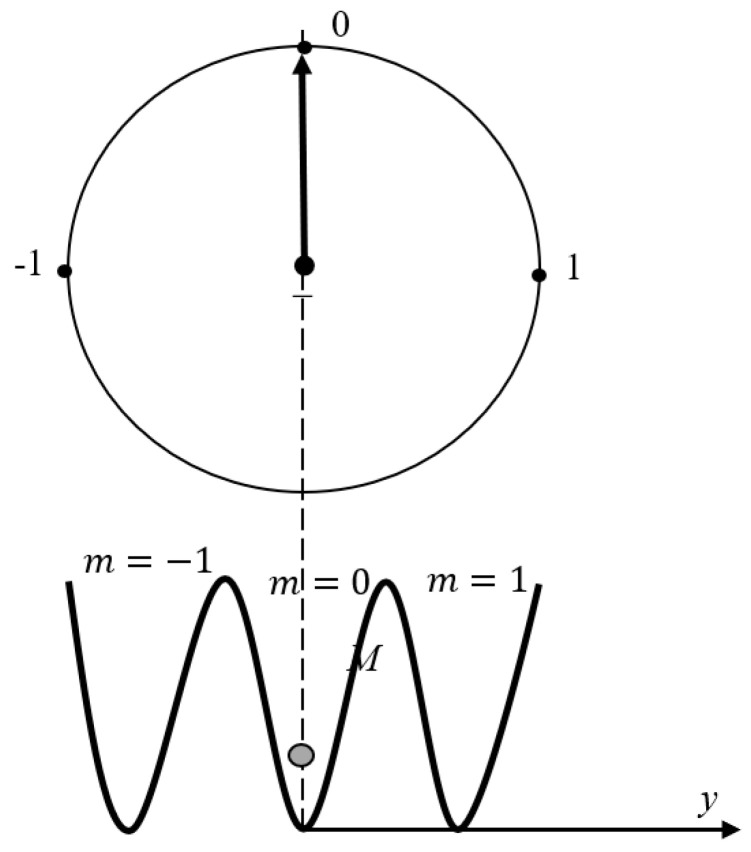
The trit-based model of a memory exploiting a Brownian particle *M* in a symmetrical triple-well potential with position *y* which can be stably trapped in either central, left or right well, corresponding to the informational states, namely: m=−1; m=0; m=1.

Now consider the computing device based on the ternary logic, and using the “trit” computing element, as presented in Figure 4 and discussed in References [1,2,3,6]. Finding particle *m* in the certain one third part of the chamber corresponds to the recording of 1 trit of information. When both the partitions are removed, the location of the particle is uncertain, which corresponds to the erasure of 1 trit of information. The analysis of the minimal Carnot engine which work is analogical to removing/introducing the partition immediately yields that the work necessary for erasing of the “trit” of information equals $W=k_{B}Tln3$. The same conclusion arises from the analysis of the “trit”, based on the Brownian particle in a triple-well symmetrical potential, analogical to that depicted in Figure 3 and shown in Figure 5. Indeed, in this case $p_{0}=p_{1}=p_{2}=\frac{1}{3}$, and again, we obtain the Landauer bound $W=k_{B}Tln3.$ It seems from a first glance that the ternary computer device is well-expected to be energetically unfavorable, when compared to the computing device, based on the binary logic. However, this conclusion is erroneous. Indeed, “trit” equals to log_2_3 bits of information [29]. Thus, an energy bound for erasing of one bit of information for the ternary computers equals:

(2) $$W_{bit}=\frac{k_{B}Tln3}{log_{2}3}=k_{B}Tln2$$

It is recognized from Equation (2) that the erasing of 1 bit of information for the ternary computer equals to that inherent for the binary-memory-based one. Generalization of Equation (2) for the *N*-based memory is straightforward:

(3) $$W_{bit}=\frac{k_{B}TlnN}{log_{2}N}=k_{B}Tln2$$

We conclude that the Landauer bound, which is necessary for erasing one bit of information $W=k_{B}Tln2$ remains the same for the computers that are based on a many-valued logic. 

## 3. Conclusions

The physical roots, justification, and precise meaning of the Landauer principle remain debatable and were exposed to the turbulent discussion recently [7,8,9,10,11,12,30,31,32]. The present paper is devoted to the very particular question: If we assume that the Landauer principle holds for the binary-logic based computing device, should it hold for the many-valued logic computer? In other words, if we adopt the principle that a minimum possible amount of energy required to erase one bit of information within a binary logic computer equals the Landauer bound $W=k_{B}Tln2$, what minimal energy should be spent for the same purpose within the many-valued-logic-based computer? Starting from the analysis of ternary-logic-based computing device [1,2,3,33], we demonstrated that the Landauer limit, necessary for erasing of one bit of information $W=k_{B}Tln2$ remains the same for computers based on a many-valued logic. Thus, the universality of the Landauer principle is shown.
